# Gas–Water Distribution and Gas Reservoirs Accumulation Properties of Upper Paleozoic Sequence in the Western Yishan Slope, Ordos Basin, NW China

**DOI:** 10.1021/acsomega.4c08407

**Published:** 2024-12-19

**Authors:** Zhen Yang, Jinsong Zhou, Jiahao Chen, Wei Cheng, Shuai Jing

**Affiliations:** † Natural Gas Research Institute of Shaanxi Yanchang Petroleum (Group) Company, Ltd., Xi’an 710065, China; ‡ Faculty of Earth Resources, China University of Geosciences, Wuhan 430074, China; § Department of Geology, Northwest University, Xi’an 710069, China; ∥ Gas Field Company, Shaanxi Yanchang Petroleum (Group) Co., Ltd., Yan’an 716000, China

## Abstract

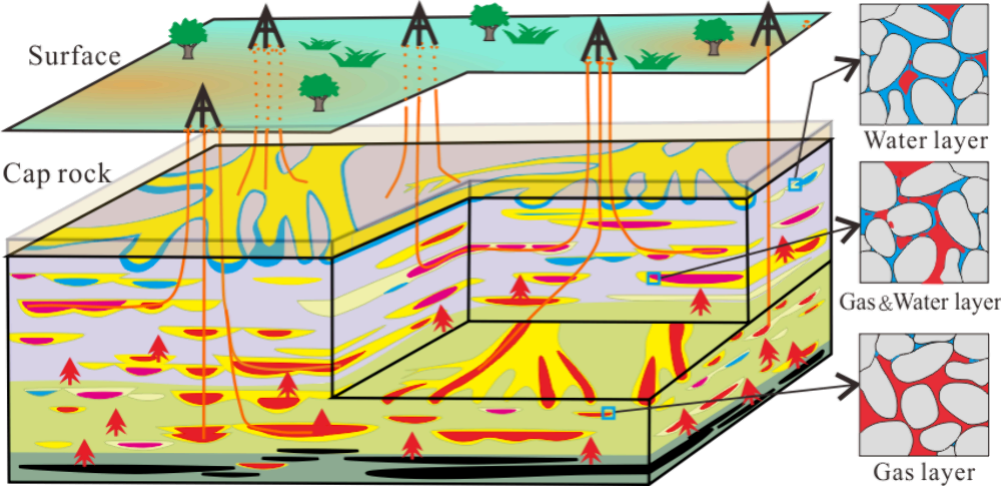

Gas–water distribution is significant in the determination of hydrocarbon accumulation mechanisms in gas reservoirs, especially for the exploitation of tight sandstone reservoirs. One of such examples are the gas reservoirs in the Yishan Slope in China, where the internal relationship between gas–water distribution is poorly understood. The pattern and controlling factors for gas–water distribution in tight sandstones gas reservoirs in the Yishan Slope have been examined from macro (such as sedimentary and anticlinal structures) and micro (such as pore throat size, heterogeneity) perspectives, using data from rock eval pyrolysis, sedimentary structure, sediment diagenesis, gas migration, mercury injection experiments, and well logs. The results showed that distribution of fluids is relatively complicated in the study area, and the gas wells and water wells are distributed across the plane. These wells are with no obvious boundaries; In the vertical plane, the Shan 1 Formation penetrated the gas layer, and water production gradually increases from the Shan 1 Formation to the He 8 Formation. The gas–water distribution is subject to hydrocarbon-generating intensity and diagenesis, while microscale physical properties and pore-throat structure of the reservoir also have a significant impact on the distribution. The Shan 1 gas reservoir is adjacent to the Taiyuan source rock and has a high hydrocarbon charge potential, which in turn drives out the formation water to form the main gas-bearing formation. In addition, areas with significant diagenetic imprints, such as strong dissolution and weak cementation, are also favorable locations for gas accumulation due to favorable physical properties and pore connectivity. Burial history and hydrocarbon generation and expulsion history showed that there are two periods of gas accumulation (175–200Ma; 105–140Ma), source rocks had reached a midhigh maturity phase, and a large amount of kerogen was generated and expelled, with natural gas transported and charged through the transport system during the second period. Hydrocarbon generation intensity essentially determined the volume of gas accumulated in the reservoir; longer continuous charging is more conducive to the formation with high gas content. This study identifies and discusses some of the controlling variables for gas–water distribution and predicts potential exploration targets for tight gas sandstone reservoirs.

## 1 Introduction

Dedicated studies on gas–water distribution in tight sandstone gas (TSG) reservoirs are essential for the development these reservoirs, as previous conventional formation water studies may not fully describe the complex geological conditions in tight sandstone reservoirs.^1−3^ TSG reservoirs are plagued with high water production and complex gas–water relationship—both of which are key to the development of gas reservoirs.^4−6^ In conventional gas reservoirs, gas is mostly sitting above the water due to buoyancy pressure.^7^ The distribution of fluids is greatly affected by the distance between the source rocks and the reservoir, hydrocarbon generating potential, and the sealing capacity of the caprock.^8,9^ In addition, reservoir properties (such as porosity and permeability) also control the gas–water distribution at the microscopic scale.^10,11^ However, the circulation of internal fluids in TSG is complicated and the abnormal phenomenon of gas–water inversion sometimes occurs.^12^

Previous evidence indicates that gas–water distribution in TSG is the result of multiple factors including tectonic evolution, hydrocarbon source rock potential, reservoir physical properties, and fracture dynamics.^13,14^ Massimo et al. suggested that the major factor that controls gas–water distribution is structure and reservoir physical properties.^15^ Gas in reservoirs is mostly concentrated on structural highs, mostly juxtaposed against traps, as seen in most gas reservoirs in the Ordos Basin.^16,17^ Higgs et al. found that the properties of TSG are affected by the deep burial process, with obvious mechanical and chemical compaction, which is significantly dependent on the porosity formed by mineral alteration and results in gas saturation being positively correlated with reservoir porosity.^18^ The presence of fractures in sandstone reservoirs have a significant impact on fluid migration pathways and plays a crucial role in the evolution of gas–water distribution.^19,20^ Different source rock formations may have significant differences in gas production irrespective of the source rock properties.^21−24^ In addition, TSGs have strong heterogeneity and relatively low physical properties, which lead to disproportionate gas reservoirs.^25−27^ Recently, significant progress had been made in TSG research; however, these studies are limited to a single controlling factor, while ignoring the fact that the influence on the gas–water distribution may be multifaceted.^28^

Western Yishan (WY) in the Ordos Basin is the most valuable gas base in China.^29^ However, high water production and a complicated gas–water relationship have impaired the exploration and development process,^30,31^ although many reports have documented the features of formation water and the diagenesis of reservoir rocks as well as gas–water distribution in strata.^32,33^ However, the main controlling factors and accumulation process remain unclear. Hence, we investigated the following: (1) the macrocontrolling factors for gas–water distribution in TSG reservoirs such as hydrocarbon generation intensity, structure, sandstone distribution, and diagenesis; (2) microcontrolling factors that contribute to gas–water distribution in TSG reservoirs such as reservoir physical properties, pore-throat structure, and migration index; (3) natural gas accumulation processes [there are two periods of gas accumulation (175–200Ma; 105–140Ma), hydrocarbon generation intensity essentially determined the volume of gas accumulated in the reservoir, longer continuous charging is more conducive to the formation with high gas content, and gas reservoir is typical of a system that is charged during cementation].

## 2 Materials and Methodology

### 2.1 Geological Setting

**Figure 1:**
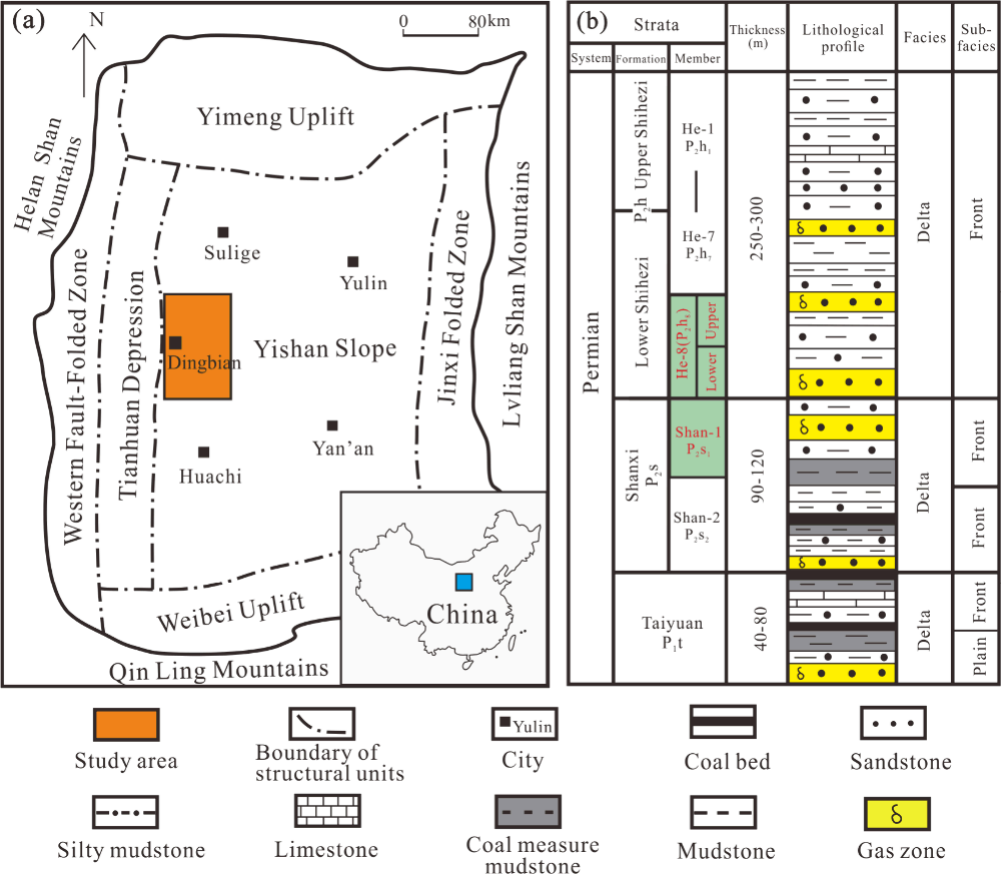
(a) Location of the study area and tectonic units of the Ordos Basin. Adapted in part with permission from ref (35). Copyright 2022 Acta Sediment Sin. (b) Stratigraphic burial history. The studied He 8 Member and Shan 1 Member are marked in red.

The Ordos Basin is located in central China, to the west of the Lvliang Mountains and east of the Helan Mountains.^34^ The Ordos Basin can be divided into six tectonic settings, namely, the Yimeng uplift, the western fault-folded zone, the Tianhuan Depression, the Weibei uplift, the Jinxi folded zone, and the Yishan slope, of which the Yishan slope is a gently sloping morphology (Figure 1a).^35^ The slope and adjacent Tianhuan Depression areas are considered primary gas reservoirs in the basin.^36^

The study area, the WY Slope, is in the midwestern part of the Ordos Basin and covers approximately 10000 km^2^. It is adjacent to Sulige gas field in the north and connected with the Tianhuan Depression in the west. The upper Paleozoic strata in the WY Slope is a sequence of clastic rock sedimentary systems with marine and continental transition facies.^37^ The Permian strata are successively developed into Taiyuan Formation (P_1_t), Shanxi Formation (P_2_s), and Lower Shihezi Formation and Upper Shihezi Formation (P_2_h), with a total deposited thickness of roughly 500 m. The source rocks are essentially coal beds and dark mudstone of the Taiyuan Formation and Shanxi Formation, which have the characteristics of being extensively hydrocarbon-generating.^38^ TSG is derived from the Shanxi and Lower Shihezi Formations.^35^ The first to seventh Members (He 1 to He 7) of the P_2_h are the regional cap rocks. The main gas producing strata are the first Member of the Shanxi Formation (Shan 1) and the eighth Member of Xiashihezi Formation (He 8), among which He 8 can be further subdivided into Upper and Lower He 8. The Shan 1 Formation developed as shallow meandering-river delta deposits, while the He 8 Formation developed as shallow braided-river delta deposits, with regional conformable contacts (Figure 1b).^39^

### 2.2 Reservoir Physical Properties

In order to establish the correlation between the physical properties of reservoirs and gas accumulation, we used sealed core drilling methods to obtain cores and perform correlation analysis.^40,41^ A total of 148 rock samples from 35 wells were selected for test analysis (29 samples from Upper He 8, 82 from Lower He 8, and 37 from Shan 1 Member were randomly selected). The selected core samples were placed into the gas gathering device and immediately vacuum sealed for 5 min. This was followed by degassing for 72 h, and the amount of vacuum is recorded alongside collection of the extracted gas and water. The distillation extraction method is used to extract all bound water and remaining movable water from the dissolved samples, and gas saturation of samples is calculated by eq 1 and eq 2.
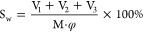
1
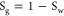
2where V_1_ is the volume of water collected by the core after decompression, V_2_ is the volume of water separated by the distillation of gas, V_3_ is the volume of water collected during the last distillation of core, and M is the weight of the dry core sample. φ is the effective porosity, S_w_, water saturation, and S_g_, gas saturation.

The porosity and permeability of the samples were analyzed by an overburden pressure porosity permeability measuring instrument PoroPDP-200.

### 2.3 Rock Eval

A total of 85 source rock samples (23 mudstones and 62 coal samples, among them 34 samples from Taiyuan and 51 from Shan 2 Formations) were collected from Taiyuan and Shan 2 Formations for further study. The organic carbon content of source rocks was tested by a Rock-Eval 2 analyzer (France). The samples were crushed and passed through 80 mesh sieves. Finally, samples were put into the high-temperature oxygen flow for combustion, and total organic carbon was converted into CO_2_. The total organic carbon content (TOC) was detected by an infrared detector. The pyrolysis analysis was performed under the test conditions of 300 °C constant temperature for 3 min to liberate free hydrocarbon as S_1_. This was followed by a programmed temperature rise of 50 °C/min from of 300 to 600 °C; then we got pyrolysis hydrocarbon S_2_. We burned these processed samples to obtain S_4_. Finally, TOC can be calculated from TOC = 0.83 × (S_1_ + S_2_ + S_4_).

### 2.4 Hydrocarbon Generation Intensity Calculation

The hydrocarbon generation intensity integrates all parameters related to hydrocarbon generation capacity source rocks and is calculated using (eq 3).^42,43^
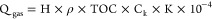
3where Q_gas_ is hydrocarbon generation intensity, ×10^8^ m^3^/km^2^; H is the source rock thickness, m; ρ is the strata density, g/cm^3^, ρ_mud_ = 2.6 g/cm^3^, ρ_coal_ = 1.55 g/cm^3^; TOC is the residual organic carbon content, wt %; C_k_ is the organic carbon recovery coefficient, 1.5;^33^ K is the hydrocarbon generation rate, mL/g_TOC_, K_mud_ = 120 mL/g_TOC_, K_coal_ = 265 mL/g_TOC_.

### 2.5 Petrographic Analysis

Thin sections were impregnated with blue-dye resin, and a Zeiss Axio Scope A1 microscope was used to observe thin slices (n = 435, 113 samples from Upper He 8, 201 from Lower He 8, 121 from Shan 1 Member were randomly selected) and count points (450 points per slice) to quantify the clastic skeleton particles, authigenic minerals, and interstitial materials. The types of pore space, distribution characteristics, diagenetic stages, and the development of dissolved pores were identified.

### 2.6 Mercury Injection

Pore throat structures of samples were tested by an ASPE-730 constant velocity porometer. Mercury was injected into samples at a quasi-static rate (5 × 10^–5^ mL/min), which ensured that the interfacial tension (480 mN/m) and contact angle (140°) remained unchanged during the experiment. A high-precision pressure sensor was used to record the change in pressure with the amount of mercury injection during the experiment, and a mercury injection curve was generated. When the maximum pressure set by the experiment was reached, the mercury inlet valve was shut and left to stand for 15 min before mercury extraction took place. The pressure changes with the mercury extraction during the experiment were recorded through the sensor until no further changes in volumes are observed. In addition, according to the capillary force formula, the capillary radii corresponding to different pressures during the experiment were calculated and the columnar chart of pore size distribution was obtained by (eq 4).^44^
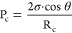
4where P_c_ is capillary pressure, Pa; σ is interfacial tension, 480 mN/m; θ is wetting angle, 140°; and R_c_ is pore radius, μm.

### 2.7 Migration Index

The migration distance of gas is determined by the change in hydrocarbon components as a result of the conditions along the migration path.^45,46^ For the components with the same molecular weight (such as iC4 and nC4), the force on the surface of mineral rocks with low effective molecular diameter (such as iC4 = 5.278 Å) is relatively weak, while the force on the surface of mineral rocks with large effective molecular diameter (such as nC4 = 5.784 Å) is relatively large.^47^ The diffusion coefficient of iC4 is also higher than that of nC4. Therefore, the iC4/nC4 ratio shows an increasing trend in migration direction, with the increase of migration distance, R_3_ increases and R_4_ decreases, making the migration index (ΔR_3_) show an increasing trend as shown in eq 5.
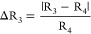
5where R_3_ = iC_4_/nC_4_ and R_4_ = iC_4_/nC_3_.

A total of 120 gas samples were selected from Shan 1 and He 8 reservoirs (among them 30 samples from Upper He 8, 61 from Lower He 8, and 29 from Shan 1 Member were randomly selected). The components were detected by a CP-4900 gas chromatograph with a scanning rate of 1250u/s and an average consumption of 5ul of each gas sample. The samples were depressurized and dry filtered before being placed in the chromatograph. The experiment process adopted standard industrial standards as described by Yu et al.^17^

## 3 Results and Discussion

### 3.1 Gas–Water Distribution Characteristics

#### 3.1.1 2D Distribution

**Figure 2:**
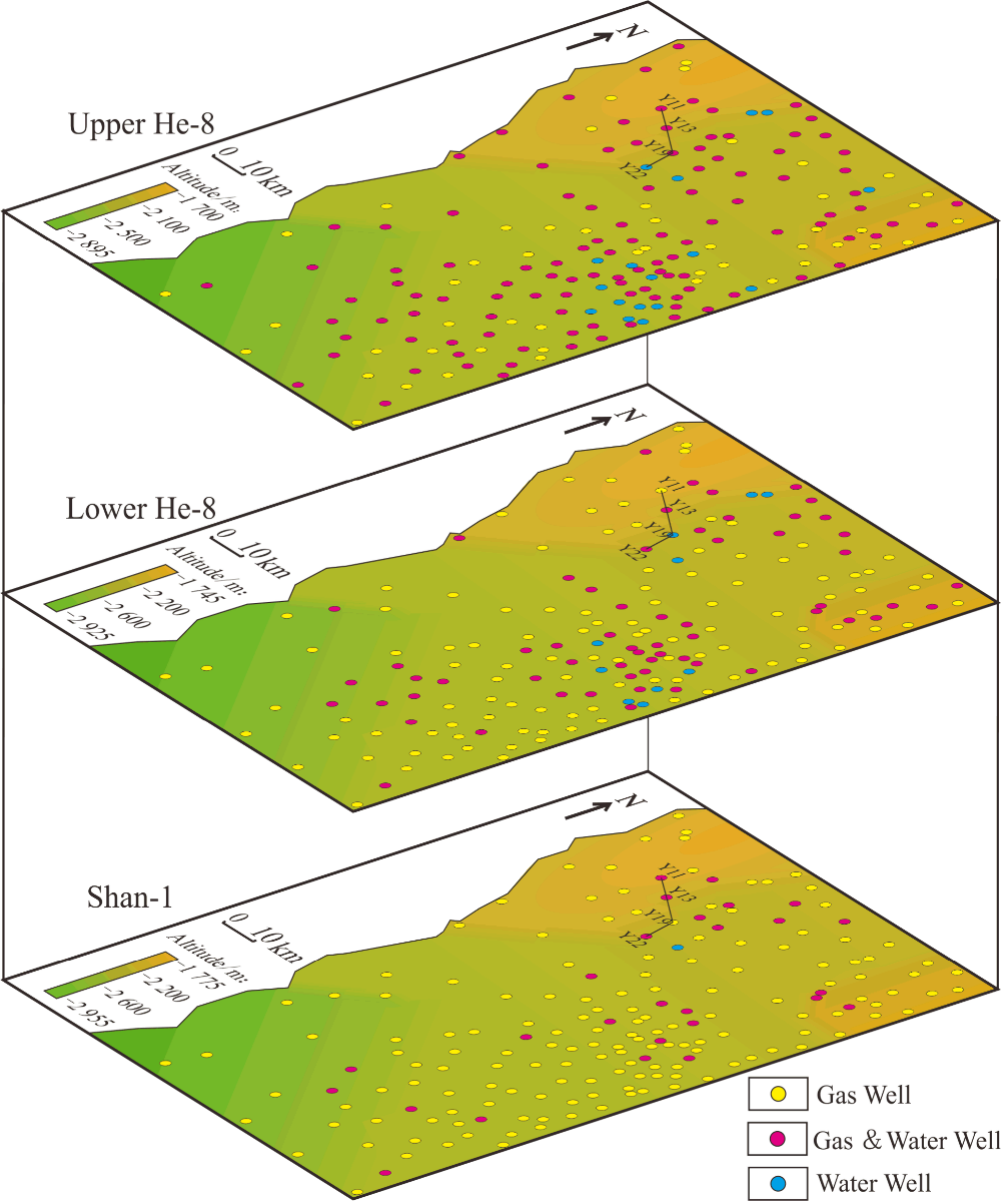
2D distribution of gas wells and water wells in Shan 1–He 8.

From our study, we observed that the percentage of gas wells is the highest, accounting for 84% in Shan 1, and that water wells are few and scattered (Figure 2). In the Lower He 8, gas wells accounted for 59%, gas–water wells accounted for 35%, and dry wells accounted for 6%. Water wells are mainly scattered in the central and northern parts of the study area. The number of gas wells is the lowest in the Upper He 8, accounting for 29%, while gas–water wells and water wells are distributed throughout the region. From Shan 1 to He 8, the gas wells and water wells are distributed across the plane without obvious pattern.

#### 3.1.2 Vertical Distribution

**Figure 3:**
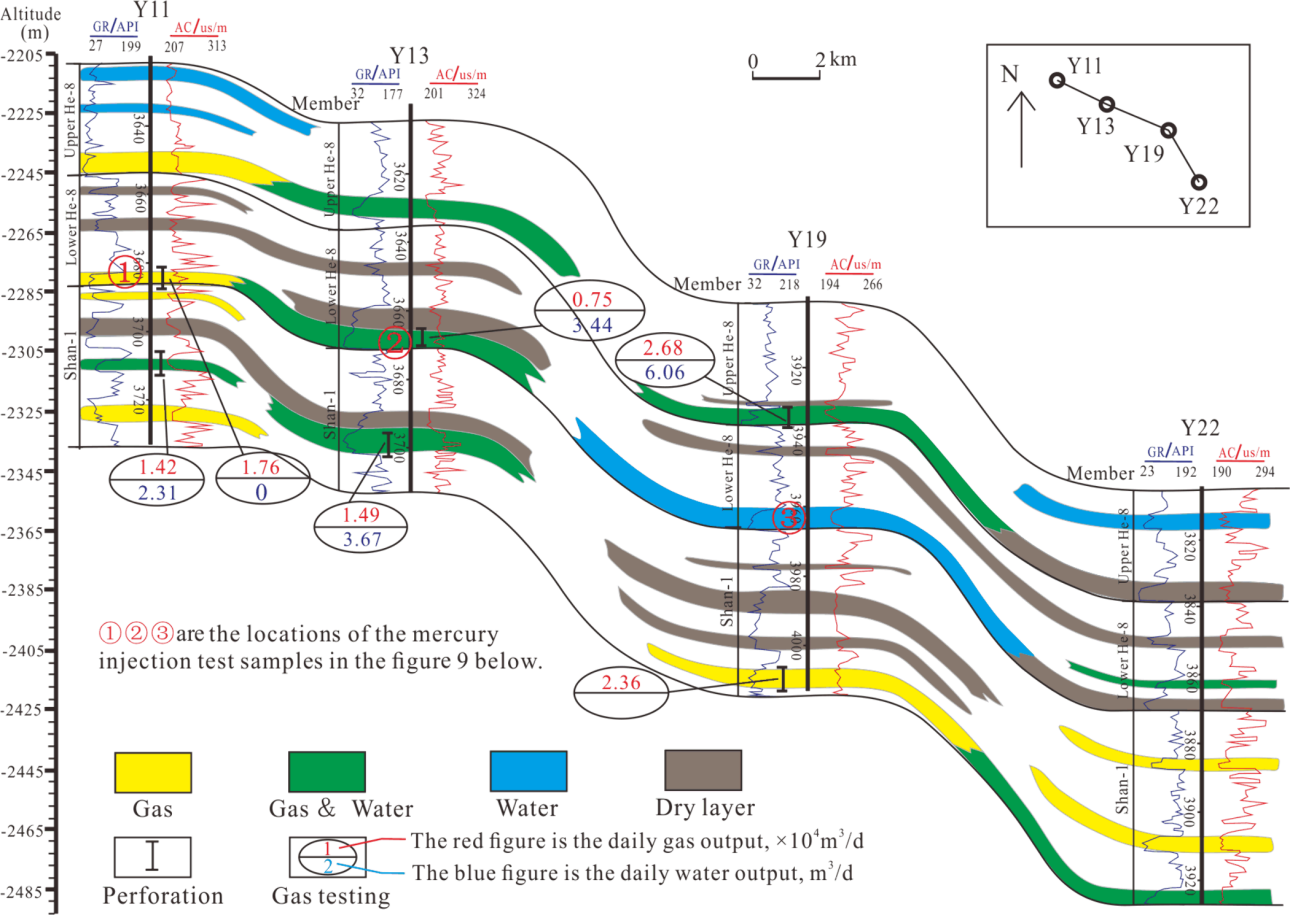
Gas reservoir profile of the Y11–Y22 well in Shan 1–He 8 (the location of the connecting well line is shown in Figure. 2). Test samples shown in Figure 9 came from areas labeled “1”, “2”, and “3”.

The presence of mudstone and tight sandstone in the formation resulted in the complex gas–water distribution with no obvious and uniform demarcation interface. However, the lateral connectivity of each sand body presented a pattern of upper gas and lower water intervals (Figure 3). The horizontal and vertical gas–water distribution characteristics showed that the water layer is not controlled by regional structure and no uniform boundary distinction between the gas–water layer on the plane.

### 3.2 Macro Controlling Factors of Gas–Water Distribution

#### 3.2.1 Hydrocarbon-Generating Intensity

**Figure 4:**
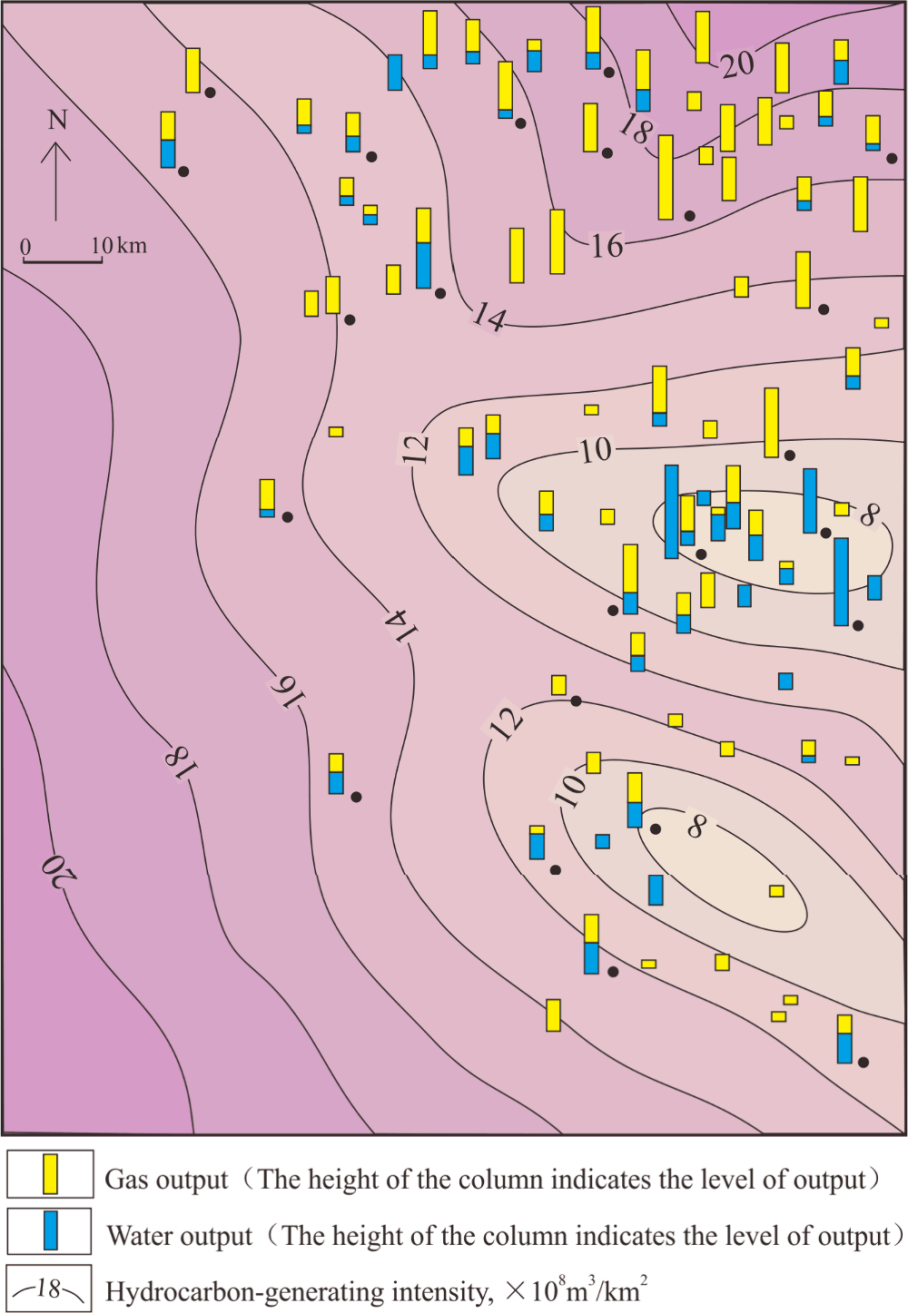
Hydrocarbon generation intensity of the Upper Paleozoic and gas production of the Lower He 8 reservoirs.

Hydrocarbon generation intensity is the amount of hydrocarbon generation of source rocks per unit area, which is related to the thickness of source rocks, the abundance, type, and maturity of organic material.^48,49^ We calculated the hydrocarbon generation potential through geochemical parameters and the thickness of coal seams and dark mudstone. The central and southern parts of the study area have relatively low value in terms of hydrocarbon generation intensity, while the northern part has relatively high values, with the hydrocarbon generation intensity reaching 20 × 10^8^ m^3^/km^2^ (Figure 4).

The Shan 1 reservoir is closer to source rocks and mainly produces gas, while the Upper He 8 produces high volumes of water, which suggests that hydrocarbon generation intensity partly controls the gas–water distribution vertically. From the distribution of wells in the study area, water production was common in the areas with low hydrocarbon generation intensity in the southeast, while high gas production was associated with high hydrocarbon generation intensity in the northeast. This suggests that hydrocarbon generation intensity is one of the main controlling factors of the gas–water distribution in this area.

#### 3.2.2 Small-Scale Structure

Structure plays an essential role in controlling the migration and accumulation of gas. The gas reservoirs have poor lateral continuity, with gas layers, gas–water layers, and water layers mostly intersecting and superimposed (Figure 2). However, we can see that the well located on a structural high surface (Figure 3, Y11) showed gas production in Shan 1 and He 8 reservoirs. The gas layer of the Shan 1 Formation exists in isolated sand bodies cut off by tight sandstone layers in the downdip direction. On the other hand, the gas layer in the Lower He 8 was connected with the gas–water layer in the downdip Well Y13, and the same is true in the Upper He 8, which suggested Well Y11 has good reservoir-forming conditions. The Upper and Lower He8 of Y19 are both trapped within tight sandstone and mudstone units, as well as in the downdip direction, leading to low gas migration, while Shan 1 Formation in well Y19 was located in a structural high of a connected sand body, resulting in high gas accumulation. We also can see that well Y22 located in the structural low state produces gas/water (Figure 3). Based on exploitation practice of this gas reservoir, the upper part of the connecting sand body consisted of gas only, while the lower part consisted of a gas–water layer. This suggests that the distribution of gas and water within this sand body is primarily subject to lithology and structure.

#### 3.2.3 Configuration of Sand Bodies

**Figure 5:**
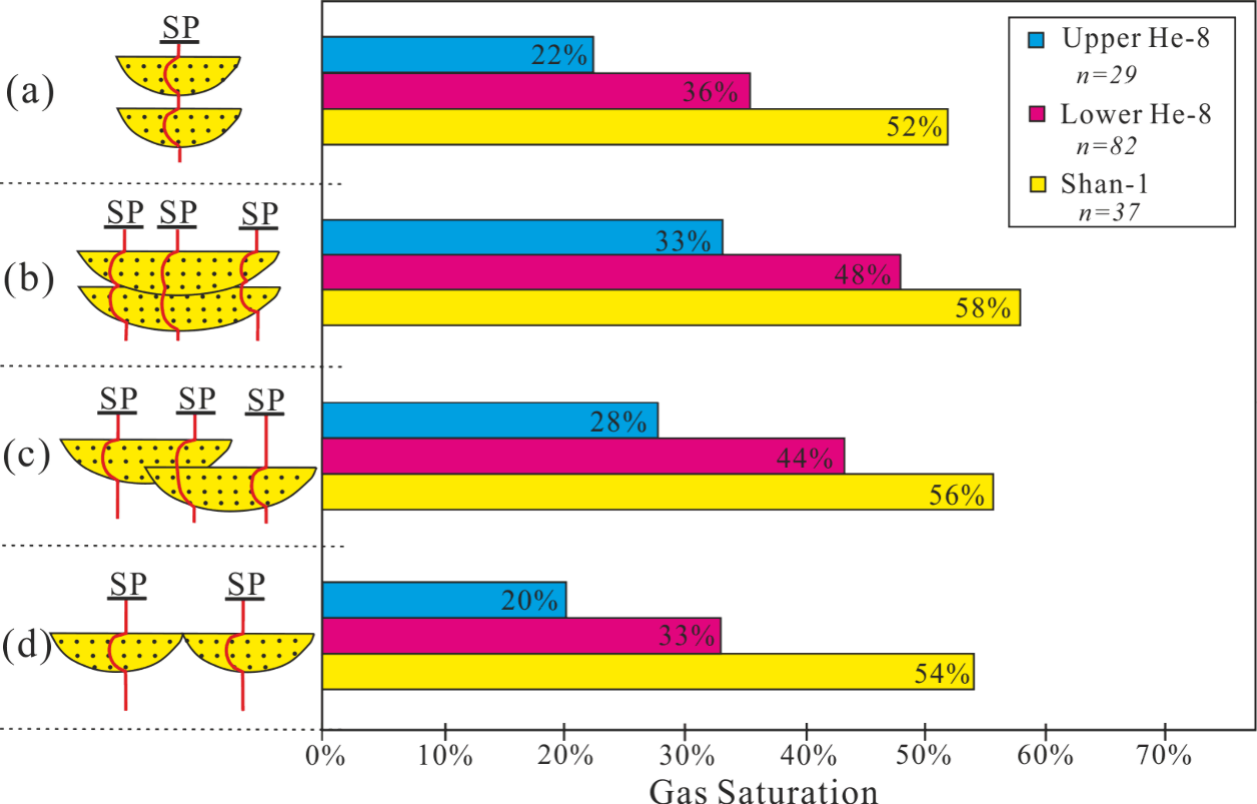
Relationship between different sand body patterns and gas saturation: (a) isolated sand body, (b) vertically superimposed sand bodies, (c) laterally tangential superimposed sand bodies, and (d) horizontally bridged sand bodies.

The superimposed relationship of sand bodies from Shan 1 to He 8 in the WY is divided into four main types: isolated, vertically superimposed, laterally tangential superimposed, and horizontally bridged sand bodies (Figure 5). The isolated sand bodies generally represented a single channel that underwent rapid deposition.^50^ The vertically superimposed sand bodies showed a weak swing in amplitude of a single channel where sediment supply is sufficient and stable, while a strong oscillation will evolve to a laterally tangential superimposed sand body.^51^ The horizontally bridged sand bodies represent a distributary channel with changing flow direction in multiple periods, but the duration of a single stage channel and sediment deposition time is relatively long.^52^ The superposition relationships represented gas accumulation conditions, leading to different gas and water enrichments in sand bodies.

In this study, vertically superimposed and laterally tangentially superimposed sand bodies showed high gas saturation. This suggests that both sand bodies have good lithophysical property combination, due to the scouring and superposition of multilevel distributary channels.

Although vertical and lateral connectivity of the isolated sand body is poor in Shan 1, it is adjacent to source rocks with a sufficient gas supply and good sealing property, which is conducive for successful accumulation of natural gas. Therefore, distribution of sand bodies is a crucial factor influencing gas–water distribution as shown in Figure 5.

#### 3.2.4 Diagenesis

**Figure 6:**
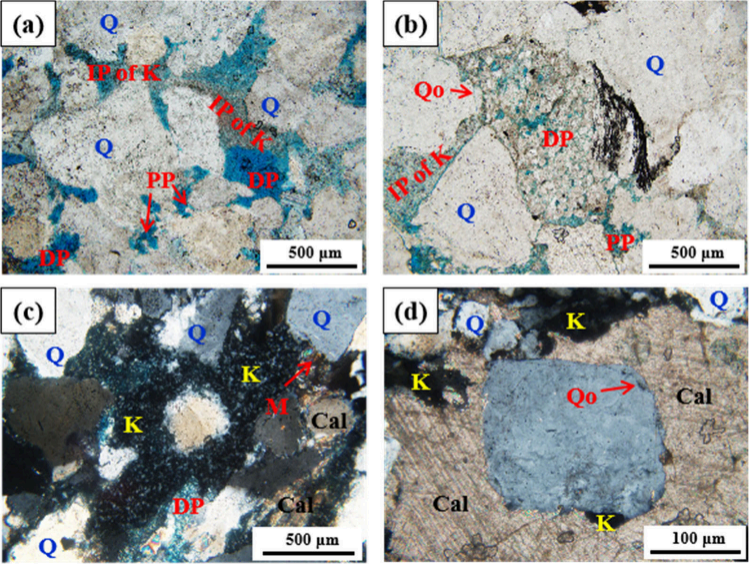
Photomicrographs showing of different diagenetic facies in He 8 Member: (a) Class I diagenetic facies (dissolution intensity > 20%; cementation intensity < 5%), intercrystalline pores (IPs) of kaolinite (K), dissolution pores (DP), and primary pores (PP), Well Y12, 4344m, cross-polarized light (xpl). (b) Class II diagenetic facies (dissolution intensity > 15%; cementation intensity < 10%), carbonate replaced quartz overgrowths (Qo) and quartz (Q) grains, DP, and PP, Well Y14, 3547m, xpl. (c) Class III diagenetic facies (dissolution intensity < 10%; cementation intensity > 15%), carbonate cements and K filled within pores, and late DP (note the mica (M) distortion), Well Y15, 3862m, xpl. (d) Class IV diagenetic facies (dissolution intensity < 5%; cementation intensity > 20%), carbonates filled within IP and substituted Q, K substituted carbonates, Well Y18, 3602 m, xpl. Cal = carbonate (calcite).

Pore structure and reservoir properties were controlled by lithofacies type and diagenetic modifications.^53^ Diagenetic imprints in Shan 1 and He 8 units (Figure 6), showed that the influence of compaction, dissolution, and cementation is common in the region. This greatly determines rock porosity and permeability as shown in Figure. 6.

**Figure 7:**
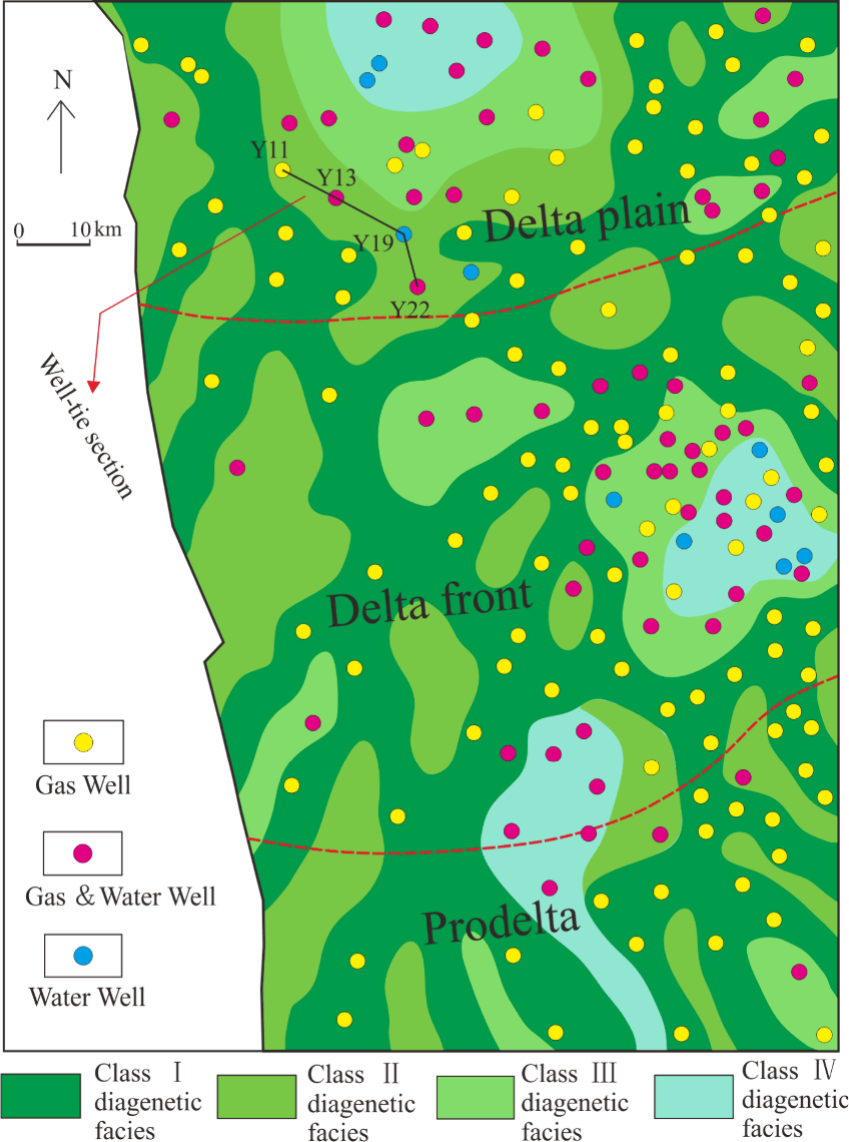
Diagenetic facies and gas–water distribution of the Lower He 8 Member.

Diagenetic facies refer to the comprehensive product of the original sediment as a result of sediment adjustment to diagenesis and its evolution in the burial diagenetic environment. This reflects comprehensive characteristics of rock particles, cements, fabrics and pores and fractures.^54,55^ According to the diagenetic characteristics of this area, the reservoir can be classified into four types of diagenetic facies (Figure 7): Classes I, II, III, and IV. The effects of dissolution gradually weakened while the effects of cementation in turn increases from Class I to IV. The results indicated that gas is mainly distributed in regions with Class I and II diagenetic facies, while dry-water wells are mostly concentrated in the regions with Class IV diagenetic facies. Hence, we concluded that diagenesis controlled the pore construction of the reservoir and is also one of the key controlling factors for the distribution of gas and water.

### 3.3 Micro Controlling Factors of Gas–Water Distribution

#### 3.3.1 Porosity and Permeability

**Figure 8:**
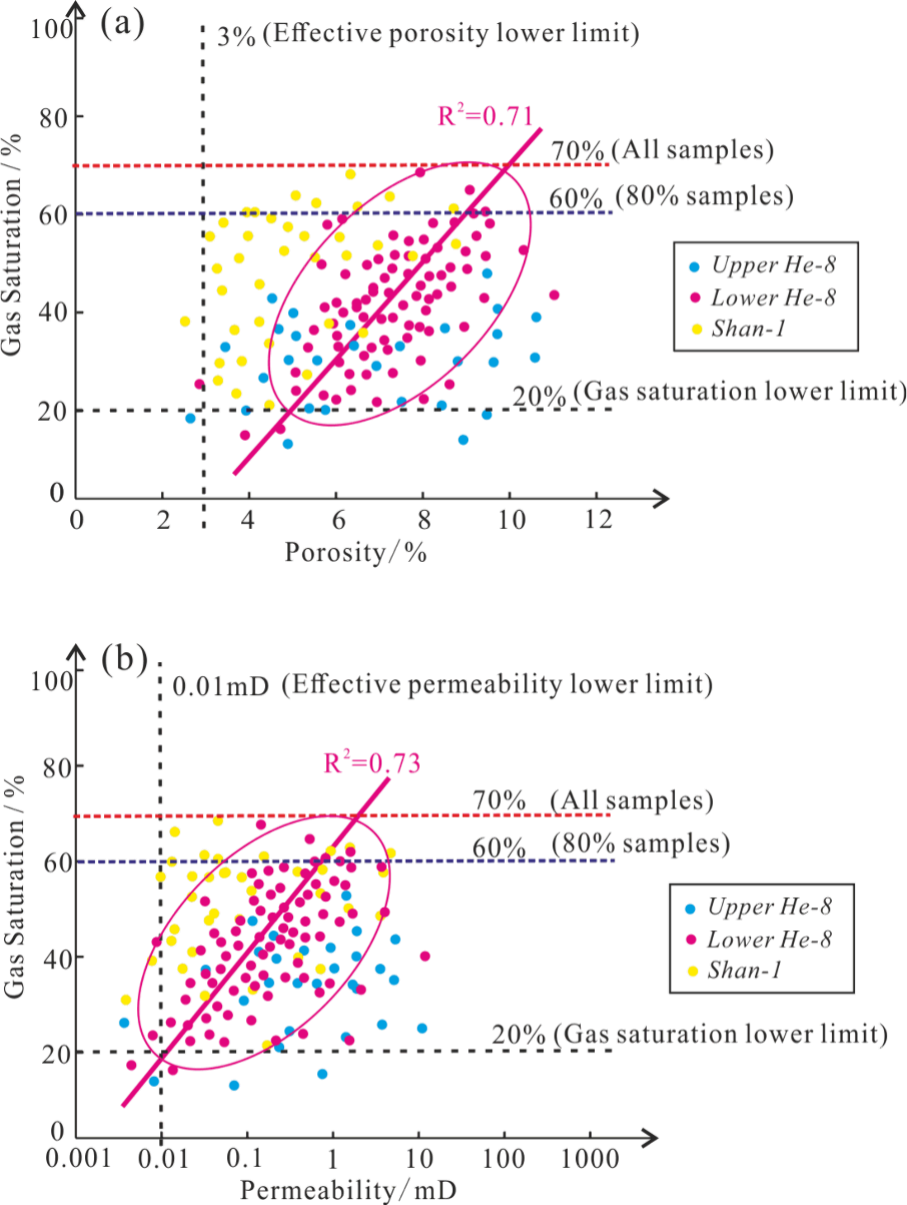
Relationship between porosity (a), permeability (b), and gas saturation of reservoir in Shan 1–He 8 Members.

Sand bodies provide migratory pathways as well as the gas storage units for the Upper Paleozoic gas reservoirs. The correlation between physical properties of the Upper He 8 and Shan 1 is weak, while the Lower He 8 shows a positive correlation, which illustrates gas saturation increases with the increase in reservoir porosity and permeability (Figure 8).

Although the sand bodies of Shan 1 were poorly connected, it generally has good gas bearing potential due to its proximity to source rocks and sufficient gas supply. On the contrary, Upper He 8 sand bodies showed good physical properties, but insufficient gas supply as a result of migratory distance between the source rock and the reservoir. Lower He 8 reservoir is at a moderate distance from the hydrocarbon generation center compared with Upper He 8. When the hydrocarbon source supply and reservoir sealing are not much different, the greater the porosity and permeability, the higher the gas saturation.

#### 3.3.2 Pore-Throat Structure

**Figure 9:**
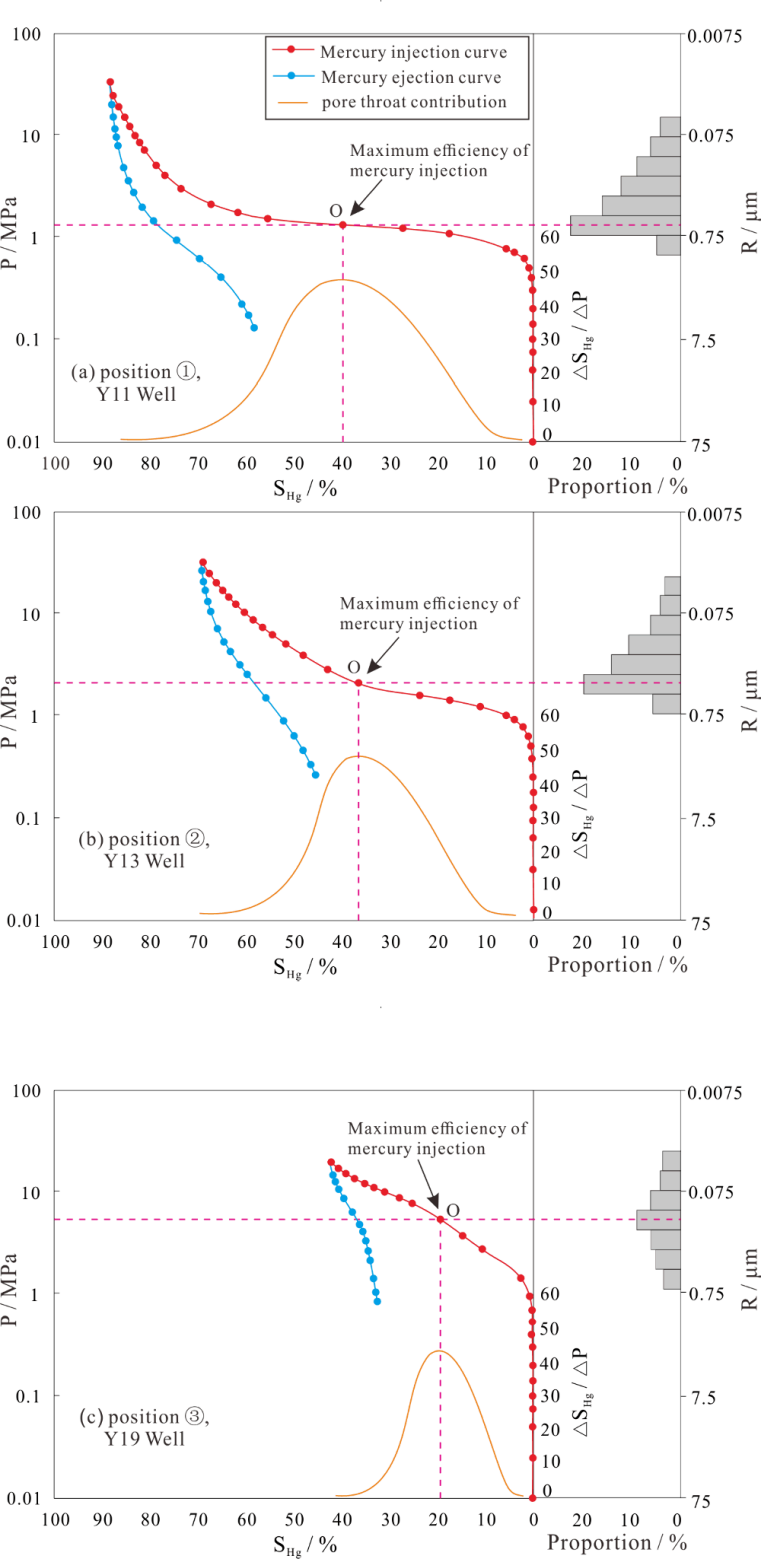
Mercury injection curve of the Lower He 8 Member reservoir (see Figure 3 for sampling location).

In this study, there is an insignificant difference in hydrocarbon generation intensity and diagenesis in this area (Figure 3), but the fluid is varied, with ① as gas, ② as gas/water, and ③ as water. We observed that local microscale structures in the study area cannot cause such distinct differences in fluid accumulation in the different well locations. Three samples from the Lower He 8 reservoir in wells Y11, Y13, and Y19 were tested for mercury injection analysis. The results illustrated that sample ① (Figure 9a) has the lowest irreducible water saturation and best connectivity and sample ② (Figure 9b) has the worst. Sample ③ (Figure 9c) showed high displacement pressure, which means poor reservoir connectivity. The areas with a complex pore-throat network have poor gas charge ability, which in turn leads to unexpelled formation water in the reservoir. Gas displaced water in the reservoirs with better pore-throat connectivity; therefore, gas wells are mainly situated in such a well-connected reservoir area. Also, in the late stage of accumulation, the occurrence of natural gas retarded the cementation and better preserved the original space of the reservoi.^30^

#### 3.3.3 Migration Index

**Figure 10:**
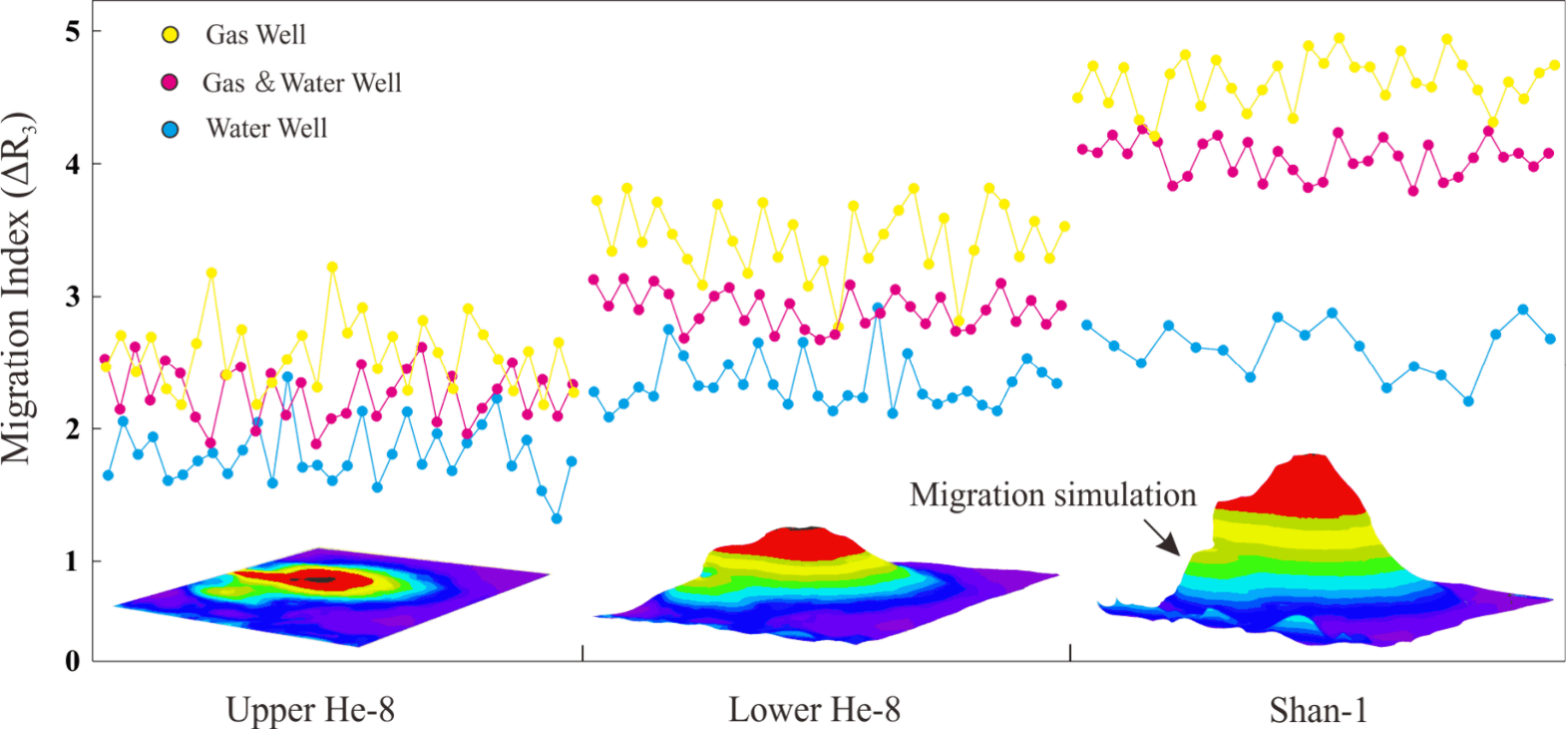
Distribution characteristics of the gas migration index (ΔR3) in the Shan 1–He 8 Members.

Migration index (ΔR_3_) is a good indicator of gas migration conditions in the study area. As the gas migration distance increases, the migration index (ΔR_3_) increases significantly. The migration index distribution in Figure 10 shows that there are more water wells in areas with a low migration index, and gas wells are largely spread in regions with a high migration index. That can be explained by the throat network of water wet reservoirs being narrow and gas–water displacement being difficult, leading to difficulty in gas migration in the water wet reservoirs.

### 3.4 Hydrocarbon Accumulation Mode

**Figure 11:**
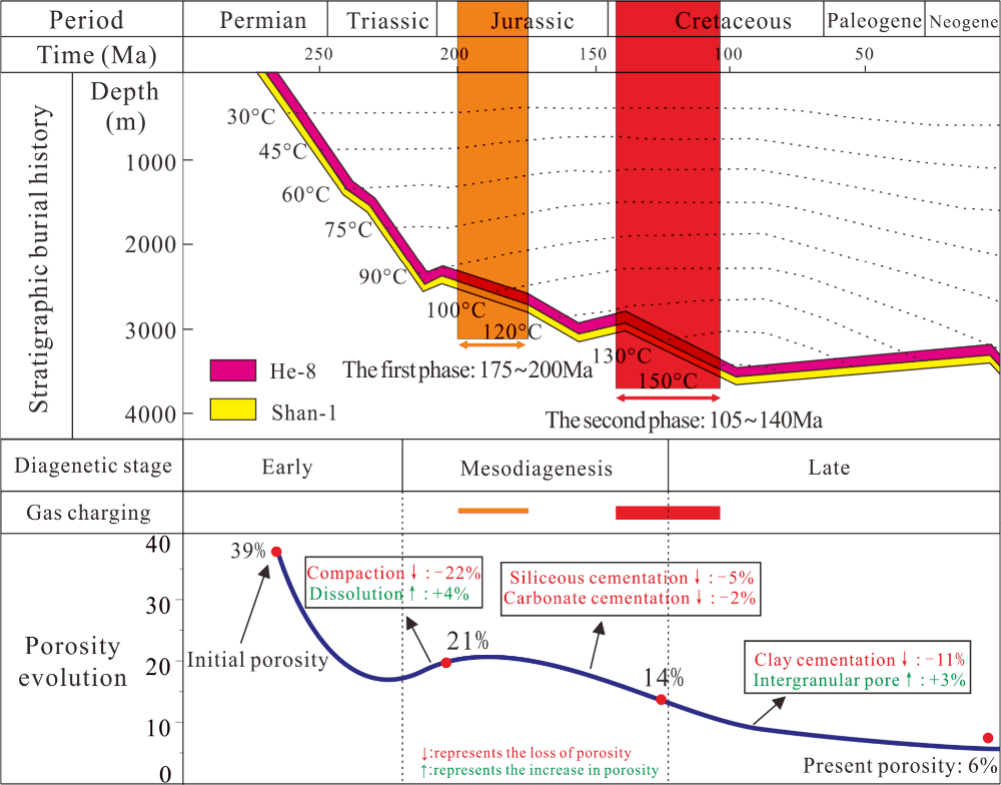
Burial history and hydrocarbon charging history of Upper Paleozoic strata in western Yishan Slope. Adapted in part with permission from ref (35). Copyright 2022 Acta Sediment Sin.

The burial history and hydrocarbon generation and expulsion history in the study area showed that there are two periods of gas accumulation (Figure 11).^56^ The first stage was the Early Jurassic 175–200Ma coal measure source rocks, which began to generate and expel hydrocarbons that migrated into the adjacent reservoirs to accumulate. At this onset expulsion stage, charging intensity was weak, with temperatures between 90 and 120 °C required for the onset of thermal transformation of kerogen. The second period occurred in the Early Cretaceous 105–140Ma, at temperature range 120–150 °C. This suggests that source rocks had reached a midhigh maturity phase and a large amount of kerogen was generated and expelled, with natural gas transported and charged through the transport system.^57^ This period represents the peak of hydrocarbon accumulation in the study area. However, this period coincides with the middle to late diagenetic stage, with strong cementation and about 6.0% porosity, which is close to the present porosity values. Therefore, the formation process of the Upper Paleozoic gas reservoir is typical of a system that is “charged during cementation”.

**Figure 12:**
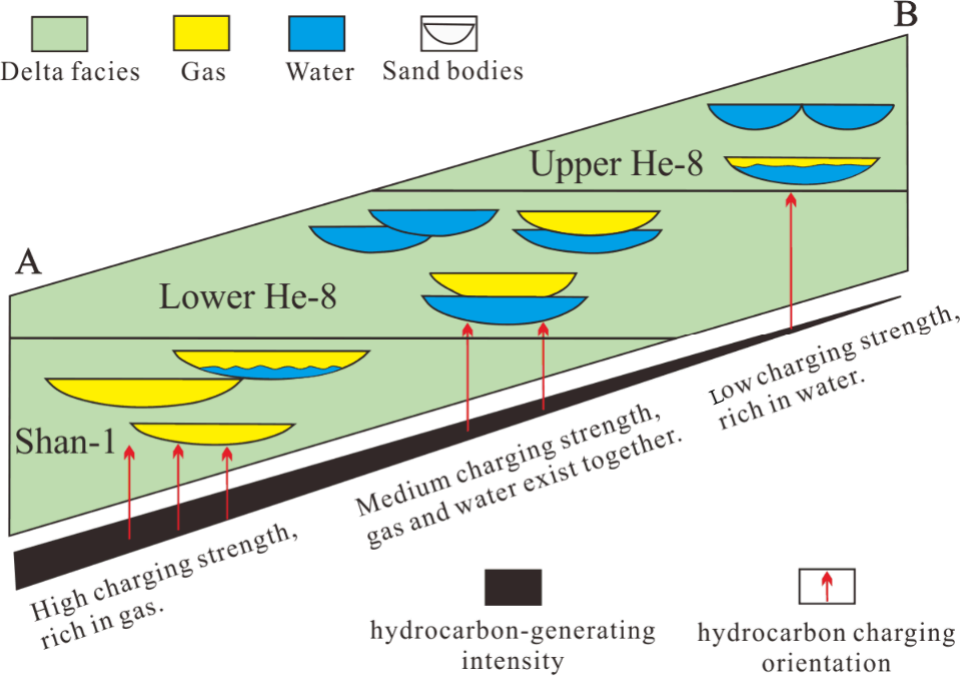
Intensities of gas source charging and genetic pattern of gas–water distribution of Upper Paleozoic strata in the western Yishan Slope.

In the Early Cretaceous, natural gas produced from the Permian source rocks accumulated in the Shan 1 reservoir and displaced the formation water that originally existed there. These reservoirs were adjacent to the underlying source rocks and had high natural gas charging intensity (Figure 12), therefore, being generally gas-bearing, even in sand bodies with poor physical properties. However, accumulation gradually decreases toward He 8, where Lower He 8 is mainly composed of gas/water and Upper He 8 reservoirs are mostly water wet. This is due to a large distance between Upper He 8 and source rock with an insufficient gas supply and weak charge. Thus, partial gas–water replacement occurs in the region.

The gas reservoirs in the study area lack edge-bottom water due to the tight reservoirs and the varied lithology and physical properties horizontally. Also, the formation is relatively flat; therefore, distinct differentiation for gas and water is nearly impossible, thus forming no distinct boundary of gas and water. In addition, the reservoirs had been relatively tight during accumulation, and formation water mostly remains in the reservoirs in the form of irreducible water, which also can interpret this phenomenon.

**Figure 13:**
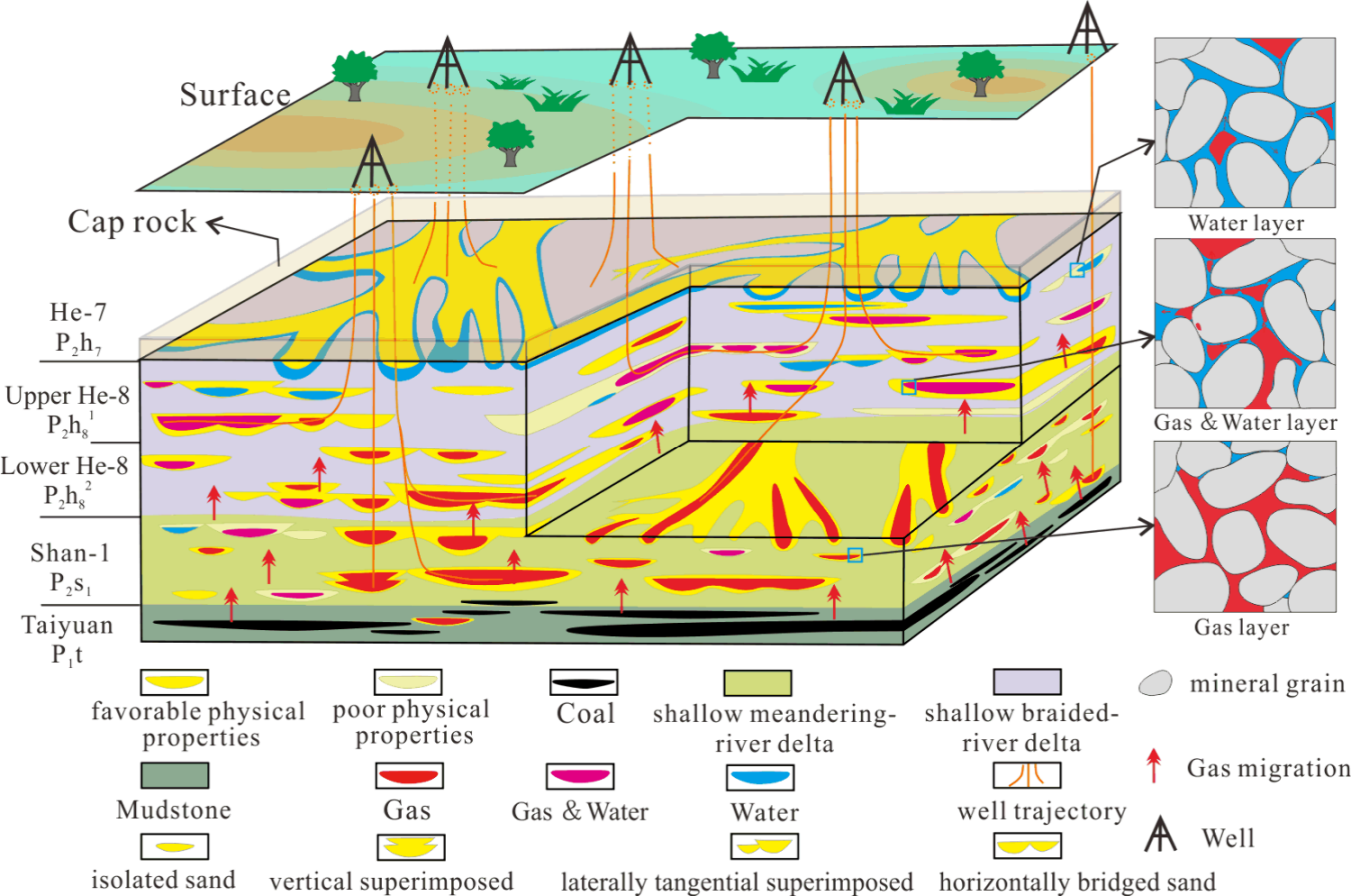
Accumulation pattern of the TSG reservoir with low hydrocarbon generation intensity in the western Yishan Slope.

Through our comparison and comprehensive analysis, we concluded that the accumulation mechanism in this TSG with low hydrocarbon generation intensity in the western Yishan Slope (Figure 13) is as follows:

(1) Hydrocarbon generation intensity essentially determined the volume of gas accumulated in the reservoir. Given that the lower gas supply in the local area with low hydrocarbon generation intensity, longer continuous charging is more conducive to the formation with high gas content.
(2) Local microscale structure contributed to gas–water differentiation in the study area.
(3) The superimposed sand bodies were scoured by multistage distributary channels and formed a good sand body, with vertically combined physical properties, which can enhance to gas enrichment.
(4) The physical properties control the gas content of in reservoirs. Gas enrichment is enhanced in reservoirs with relatively good pore-throat connectivity, while sand bodies with relatively poor physical properties exhibit poor charge ability, therefore having generally low gas content.

## 4 Conclusions

(1) Accumulation in reservoirs was subject to hydrocarbon generation intensity and diagenesis. The Shan 1 reservoir was close to source rocks and gas migrates vertically in short distance. Gas charge intensity is high and formation water is almost completely displaced by natural gas, thus forming the main gas-bearing layers. Conversely, the He 8 reservoir was at a significant distance from the hydrocarbon generation center, with insufficient gas accumulation potential. In this case more water or dry layers are generally prevalent. In addition, regions with strong dissolution and weak cementation are also favorable areas for gas accumulation due to their good reservoir physical properties and pore connectivity.

(2) The gas–water distribution was associated with reservoir physical properties and the connectivity of pore-throat during the accumulation period. The areas with better physical properties had favorable pore-throat connectivity and low capillary pressure, thus easily forming gas layers. The reservoirs with poor physical properties have complex pore-throat structure and strong capillary pressure, resulting in the retention of original formation water.

(3) Burial history and hydrocarbon generation and expulsion history showed that there are two periods of gas accumulation (175–200Ma; 105–140Ma), source rocks had reached a midhigh maturity, and a large amount of kerogen was generated and expelled, with natural gas transported and charged through the transport system during the second period. Longer continuous charging is more conducive to the formation with high gas content. Due to the infrequent development of large faults in the study area, coupled with the presence of only a limited number of small- and medium-sized faults in tectonically active regions, there is currently an insufficient seismic data foundation. Consequently, this aspect has not been thoroughly investigated; however, it aims to facilitate further discussions regarding its impact on gas reservoir distribution in future research endeavors.
